# Ten simple rules for recognizing data and software contributions in hiring, promotion, and tenure

**DOI:** 10.1371/journal.pcbi.1012296

**Published:** 2024-08-08

**Authors:** Iratxe Puebla, Giorgio A. Ascoli, Jeffrey Blume, John Chodacki, Joshua Finnell, David N. Kennedy, Bernard Mair, Maryann E. Martone, Jamie Wittenberg, Jean-Baptiste Poline

**Affiliations:** 1 DataCite—International Data Citation Initiative e. V., Hannover, Germany; 2 Center for Neural Informatics, Structures, & Plasticity, College of Engineering and Computing, George Mason University, Fairfax, Virginia, United States of America; 3 School of Data Science, University of Virginia, Virginia, United States of America; 4 California Digital Library, University of California, Office of the President, Oakland, California, United States of America; 5 Colgate University Libraries, Colgate University, Hamilton, New York, United States of America; 6 Departments of Psychiatry and Radiology, University of Massachusetts Chan Medical School, Worcester, Massachusetts, United States of America; 7 Office for Historically Black Universities Affairs, Association of Public and Land-grant Universities, Washington, DC, United States of America; 8 Department of Neurosciences, University of California, San Diego, California, United States of America; 9 University Libraries, University of Colorado Boulder, Boulder, Colorado, United States of America; 10 Department of Neurology and Neurosurgery, McGill University, Montreal, Canada; Carnegie Mellon University, UNITED STATES OF AMERICA

## Introduction

Changes in science practices are often perceived to be slow. It took about 10 years from the Collins and Tabak editorial on scientific reproducibility in 2014 [1] to see data management mandates implemented by US funding agencies [2]. However, open science practices have seen a sharp increase in adoption over the last few years, supported by policy (for example, those by the European Commission or the 2022 White House Office of Science and Technology Policy (OSTP) memo) [3,4] as well as new generations of digital tools and scientists who are embedding open values in their research practices. In this faster-paced open science environment, universities are key to fostering adoption among researchers. Universities drive implementation by advancing best practices and accounting for the needs and norms of diverse departments and disciplines. Universities are positioned to catalyze adoption of open practices through their academic evaluation processes, particularly, recruitment, tenure, and promotion. The capacity of researchers and instructors to engage with data and software scholarship will shape the next generation of students and scientists, and universities will play a crucial role in nurturing those skills by rewarding such contributions and expertise among their faculty.

The ways in which promotion and tenure committees operate vary significantly across universities and departments. While committees often have the capability to evaluate the rigor and quality of articles and monographs in their scientific field, assessment with respect to practices concerning research data and software is a recent development and one that can be harder to implement, as there are few guidelines to facilitate the process. More specifically, the guidelines given to tenure and promotion committees often reference data and software in general terms, with some notable exceptions such as guidelines in [5] and are almost systematically trumped by other factors such as the number and perceived impact of journal publications. The core issue is that many colleges establish a scholarship versus service dichotomy: Peer-reviewed articles or monographs published by university presses are considered scholarship, while community service, teaching, and other categories are given less weight in the evaluation process. This dichotomy unfairly disadvantages digital scholarship and community-based scholarship, including data and software contributions [6]. In addition, there is a lack of resources for faculties to facilitate the inclusion of responsible data and software metrics into evaluation processes or to assess faculty’s expertise and competencies to create, manage, and use data and software as research objects. As a result, the outcome of the assessment by the tenure and promotion committee is as dependent on the guidelines provided as on the committee members’ background and proficiency in the data and software domains.

The presented guidelines aim to help alleviate these issues and align the academic evaluation processes to the principles of open science. We focus here on hiring, tenure, and promotion processes, but the same principles apply to other areas of academic evaluation at institutions. While these guidelines are by no means sufficient for handling the complexity of a multidimensional process that involves balancing a large set of nuanced and diverse information, we hope that they will support an increasing adoption of processes that recognize data and software as key research contributions.

### Rule 1: Align the process to the institutional values

The institution’s values should guide its research enterprise. This includes the type and modality of scholarship that applicants are expected to demonstrate as part of academic evaluation processes. Institutions should articulate the universal principles of scholarship that it values for hiring, promotion, tenure, and other evaluations—e.g., quality, impact, and others—and document those in faculty handbooks. The departmental and college values should align to institutional values, and those collective principles should be consistently applied across institutional processes beyond researcher evaluation. Where the institution values open practices, that should be reflected in onboarding and training activities, for example, institutional programs can emphasize the importance of assigning persistent identifiers (e.g., DOIs or other persistent identifier that meets community-established best practices [7]) to all research outputs, including data and software, and of citing those contributions on their own merit in other research outputs. The institution’s commitment to open practices should also be reflected in the review of research proposals by institutional review boards. There is precedent for appointing research data experts and librarians to institutional review boards to support the evaluation of research ethics and data integrity in those domains [8].

The guidance provided in faculty handbooks should explicitly recognize that scholarly contributions are diverse, and signal that open datasets and software are eligible contributions, and expertise in those areas part of the skillset it aims to reward. Institutions should then encourage specific departments to craft guidelines aligned to those values (see examples in Box 1).

**Box 1. Examples of institutional processes, guidelines, and community initiatives that address recognition for data or software contributions**

Rule 1: Align the process to the institutional values

 •Loyola University Chicago Department of English Guidelines for Promotion and Tenure.

 •The University of Arizona Promotion Criteria by College.

Rule 2: Learn from others

 •The University of Maryland and the European Molecular Biology Laboratory (EMBL) mention data and software in the hiring, tenure, and promotion guidelines.

 •The FASEB DataWorks! Prize recognizes data sharing or reuse.

Rule 3: Tailor job postings and faculty handbooks

 •The University of Calgary guidelines for appointment, promotion, tenure, and annual assessment include a mention to data and software as part of the promotion section.

Rule 8: Articulate the metrics

 •The Make Data Count initiative has developed recommendations for the collection and reporting of data citations and normalized view and download counts for data, for example, the COUNTER Code of Practice for Research Data.

 •The NeuroMorpho.Org archive for 3D neural reconstructions provides a web-based user interface and automated alerts for reuse of data.

 •DataCite provides citation counts for open datasets and software across repositories, collected via the metadata for DOI registration, as well as data views and downloads normalized by repositories, under a CC0 license.

Rule 9: Leverage existing open tools

 •DataCite Commons: A portal that enables searches for works, people, organizations, and repositories and their connections based on persistent identifiers—works (DOI), people (ORCID), organizations (ROR), and repositories (re3data repository ID). The records in DataCite Commons provide citation counts and the citing works for individual datasets and software.

 •OpenAlex: An open-source catalog of research works; it can be a source of citation counts for data publications in journals.

 •The Data Citation Corpus: Currently in development by DataCite, the Data Citation Corpus will provide a centralized open resource that compiles data citations from a variety of sources. The corpus currently includes data citations collected via DOI metadata and identified through mining the full text of 5 million articles and preprints.

### Rule 2: Learn from others

Research assessment approaches have been the subject of much community discussion over the last several years. Some institutions have already taken steps to update their hiring, tenure, and promotion processes to recognize research outputs beyond journal publications, and include data and software. It is worth learning from those who have implemented such changes to learn what aspects may be relevant to your university or institute (examples are noted in Box 1).

In addition, some communities have developed initiatives to recognize data sharing and reuse practices. The Research Parasites awards recognize secondary analysis of data that extends, replicates, or disproves what the original investigators posited, completed in a manner that enhances reproducibility [9]. The FASEB DataWorks! Prize is an NIH-supported reward for research that showcases data sharing or reuse in biomedical fields and developed specific judging criteria for entries. These can serve as exemplars for ways in which data sharing and reuse can be framed as part of institutional guidance.

Initiatives seeking to drive reform in research assessment are also actively working to support institutional review of tenure and promotion processes, and it is worth checking their websites for resources relevant to the hiring, tenure, and promotion processes; these include DORA, the Coalition for Advancing Research Assessment (CoARA), and the Higher Education Leadership Initiative for Open Scholarship (HELIOS Open).

### Rule 3: Tailor job postings and faculty handbooks

An analysis of review, promotion, and tenure documents of a representative set of 129 universities from the United States and Canada highlighted that, among the 57 research-intensive institutions sampled, only 9 mentioned data and 37 mentioned software in their guidelines [10]. To signal that the institution values data and software contributions, it is important to explicitly invite those contributions to be listed as part of job applications and materials submitted for tenure and promotion reviews. To this end, new posts for faculty hiring should include text encouraging applicants to list data and software as part of their applications. The submission form and recommended CV template format should also have dedicated sections where applicants can list their most relevant data and software outputs. Faculty handbooks for promotion and tenure should also explicitly note that open datasets and software are recognized and include a dedicated section with guidance on how to report those contributions as part of tenure and promotion materials.

### Rule 4: Account for departmental needs

The production and use of open data varies widely across research areas, some research fields rely heavily on large collections of datasets, while others focus on a smaller number of very specific types of data. Practices for software also vary, some disciplines regularly produce software for their computational needs, while in others the use of software is less common. It is important for institutions to account for these differences and engage in discussions with faculty in order to adapt their guidelines for data and software contributions, and any metrics associated with those, to the needs of specific departments and the best practices within different communities.

Some disciplines will engage in work that produces datasets or software that cannot be shared openly, due to privacy requirements, dual-use concerns, or other restrictions. When these situations arise, there are often avenues to allow controlled release or sharing of parts of the information (e.g., metadata) about the dataset or software. The evaluation guidelines should advise candidates to outline steps taken to facilitate sharing where possible, or justify the reasons why the dataset of software has not been shared.

The framework for evaluation and the metrics that come within it will evolve as open practices advance and as the institution reviews its priorities and approach to academic evaluation. Any metrics used to evaluate individual researchers should also contribute to the overall assessment of scholarship at institutional level.

### Rule 5: Develop clear guidelines for committee members

The guidelines for the members of hiring panels and tenure and promotion committees should incorporate information to guide the assessment of candidates’ data and software contributions. To this end, the guidelines should include details on the following aspects:

- Technical competency in the creation, management, and use of data and/or software
- Innovation in the technology, design, or approaches employed to create, manage, and use data and/or software
- Service as part of professional organizations, groups, journals, or committees that support and promote open practices for data and software
- Teaching, training, outreach, and other community activities to advance best practices in open data and open-source software

### Rule 6: Select a suitable committee

Selecting a suitable committee is essential to ensure an informed evaluation of the candidate’s accomplishments. For data and software to be evaluated with equal standing to other contributions, the departmental or college committee must include members who have the expertise to assess open data and software. Institutions should be proactive in ensuring that this expertise is covered during the selection of the committee members. This can also involve an ex officio or standing committee member, and the inclusion of this member can be documented as part of the faculty guidelines for forming a review committee. If there are concerns that such expertise is not adequately covered among faculty members, consider involving reviewers from other institutions who will bring such expertise.

When requesting letters from external experts on the candidates’ accomplishments, include a specific request for comments on the candidate’s data and software contributions, referring to the institution’s recognition of those as important contributions.

### Rule 7: Ensure a dedicated review

To ensure the review of data and software is not eclipsed by other outputs, designate or appoint at least one committee member responsible for delivering an assessment of the data and software contributions listed in the candidate’s packet. Completing a review of the candidate’s contributions requires time and dedication, and committee members often find themselves strapped for time to review all the materials for the candidate pool. This dedicated review will enable the reviewer to dedicate time and focus to evaluate the quality and impact of data and software contributions specifically, and mitigate the risk of defaulting to other—potentially flawed—markers related to journal publications.

Where multiple committee members have adequate expertise, encourage them to provide comments on those areas; however, having a dedicated reviewer for data and software will ensure that the final review includes the committee’s feedback on those contributions.

### Rule 8: Articulate the metrics

Candidates should be invited to provide a narrative outline of their data and software contributions and their importance and to complement this with quantitative measures of the usage and reach of their open data and software. The guidelines for applicants should clearly indicate what metrics may be included:

#### Qualitative metrics

Applicants should be invited to provide a narrative about the impact of their data and software contributions, within their research community and beyond. This can highlight new scientific projects or activities enabled by their data and/or software (by their group or others). This section may also include information on the experimental design and protocol(s) behind the data collection, approaches to data management and organization, accuracy and timeliness of their datasets, alignment with the FAIR and CARE principles [11,12] and community standards, or other measures of quality for their data and software, such as the quality of the accompanying documentation, the use of open licenses, or whether test functionality exists for software. Guidance for applicants and the faculty handbooks should articulate what qualitative aspects of data and software contributions are relevant for the committee’s review.

#### Quantitative metrics

Data and software citations can provide a measure of their use and reuse in research (by the applicants or others), data views and downloads can signal interest in the dataset, and for software, the number of downloads and installs can point to endorsement and adoption by the community. In addition, additional measures can also provide markers of the quality of open software, such as the maturity of the software, measures of community engagement, or the speed of bug fixes.

The documentation for applicants should outline the quantitative metrics to be reported for data and software. We recommend the use of citation, download, and view counts that originate from open, nonproprietary sources and are normalized and reproducible, to ensure they are comparable across platforms. Some repositories provide users with citation counts or functionality to retrieve citations or downloads for datasets they host, e.g., the NeuroMorpho.Org archive [13]. DataCite aggregates citation metadata for open datasets and software across repositories, as well as normalized data views and downloads, and makes those metrics available to the community.

In order to ensure meaningful comparisons, it is important that measures such as views and downloads for data or software are comparable across platforms and prevent such counts to be unduly inflated by bots and web crawlers. With this in mind, we recommend that repositories and platforms that host data report normalized measures of usage per the recommendations by the Make Data Count initiative and the COUNTER Code of Practice for Research Data. These frameworks filter out usage originating from bots and web crawlers, as well as double-click activity (to prevent overcounting if, e.g., the same link is clicked multiple times in succession in response to a slow internet connection).

### Rule 9: Leverage existing open tools

**Fig 1:**
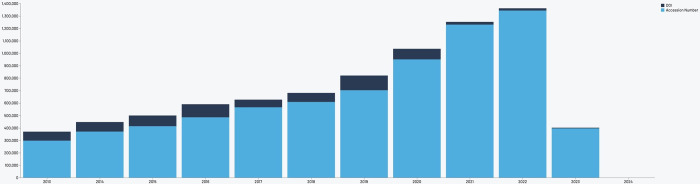
The Data Citation Corpus dashboard. Visualization of data citations over time (2013–2023) as part of the Data Citation Corpus, http://corpus.datacite.org/dashboard.

There are a number of platforms and services that provide information on citations or other measures of usage for data and software. The guidelines for applicants and for committee members should include consistent information on what tools or services are encouraged to document measures of the impact of data and software contributions, and advise applicants to always indicate the source of the information reported. Following recent developments in research evaluation (e.g., the launch of the Open Edition of the Leiden Ranking), and in alignment with the commitments set forth by the Barcelona Declaration on open research information, we recommend using existing open infrastructure that enables transparent access to the underlying metadata. Tools to gather measures of usage for data and software based on open infrastructure include DataCite Commons, a search portal for works, people, organizations, and repositories that also lists their citations, the open-source catalog of research works in OpenAlex, and the Data Citation Corpus in development by DataCite, which will provide a centralized open resource for data citations from a variety of sources (Fig 1). See Box 1 for more detail about these existing tools.

### Rule 10: Share with the community

After the implementation of a framework that recognizes data and software contributions, we recommend that the institution completes a review to determine whether the process has achieved the intended alignment with the institutional values (see Rule 1) and establish additional adjustments as needed. This is an opportunity to solicit feedback from those who participated in the process (applicants, review committee members) and to review how practices in open data and software are evolving. This enables a reflective process that can establish how the academic evaluation processes can align with community best practices. We invite institutions to share the outcomes from implementing recognition for data and software in their evaluation processes with the community, but also to communicate transparently about the difficult balance across the different dimensions of scholarly contributions that have to be considered by evaluation committees.

## Conclusions

Hiring, tenure, and promotion processes are powerful influences in driving innovation in research practices and will play a critical role in furthering adoption of open data and software. The guidelines above provide practical steps to update institutional processes to incorporate data and software and signal their recognition of those outputs on their own merit.

We acknowledge that hiring, tenure, and promotion are multifactorial processes and that data and software competency is only one of the factors that review committees need to consider across a wide range of scholarly contributions. These digital contributions need to be balanced with other factors particularly relevant for specific positions, such as the need for increased diversity, accounting for applied versus theoretical contributions, the capacity of a candidate to build collaborations, their pedagogical approach, or others. We thus acknowledge that there is no “simple rule” for how review committees can balance the different factors that inform the final hiring or tenure decision. We hope nevertheless that the items articulated above will help committees consider and value data and software contributions as a key factor in hiring and promotion.

The community best practices for research data and software will continue to evolve, and the tools and frameworks available to evaluate their use will also be subject to further development. We invite institutions who incorporate data and software into their hiring, tenure, and promotion processes to share their experiences, so that we can continue to update assessment processes towards increased alignment to open science while responding to the needs of diverse communities.
